# Neuroendocrine tumor of the lung presenting as slowly progressive, painful bilateral choroidal masses

**DOI:** 10.1016/j.ajoc.2025.102503

**Published:** 2025-12-12

**Authors:** Sharon H. Zhao, Susanna S. Park

**Affiliations:** a Department of Ophthalmology & Vision Science, University of California Davis., Sacramento, CA, USA; b Department of Ophthalmology and Visual Sciences, Illinois Eye and Ear Infirmary, University of Illinois at Chicago, Chicago, IL, USA

**Keywords:** Choroidal metastasis, Neuroendocrine tumor, Eye pain, Lung cancer

## Abstract

**Purpose:**

To report a rare case of a metastatic slow growing neuroendocrine tumor of the lung that presented as slow-growing bilateral choroidal masses with progressive vision loss and retrobulbar pain.

**Observations:**

A 64-year-old male presented with vision loss and retrobulbar pain bilaterally, progressively worsened for four months. He had no significant past medical history but had seen an ophthalmologist in Mexico starting 1.5 years earlier and was informed he may have a mass in the eye when last seen five months earlier. Ophthalmic examination revealed visual acuity of 20/400 bilaterally with large elevated amelanotic choroidal masses in both maculae with overlying pigmentary changes and subretinal fluid. The initial differential diagnosis included choroidal metastasis and infectious and inflammatory etiologies such as posterior scleritis and granuloma. Whole body PET-CT scan revealed a focus of hypermetabolic activity in the lung suspicious for a slow growing malignancy or infectious/inflammatory lesion. Due to insurance issues, there was a delay in referral to medical oncology. The choroidal lesions gradually increased in size over four months. Eventual biopsy of the lung confirmed the diagnosis of a slow-growing neuroendocrine tumor of the lung. Systemic treatment with octreotide, Capecitabine, and Temozolomide was initiated with regression of choroidal tumors, improvement in visual acuity, and resolution of eye pain.

**Conclusions and importance:**

We describe a rare case of a bilateral choroidal metastasis from a slow growing neuroendocrine tumor of the lung that presented with slowly progressive bilateral vision loss and eye pain. Despite delays in diagnosis and treatment, eye pain resolved, and vision loss improved with systemic therapy alone.

## 1 Introduction

Metastasis is the most common uveal malignancy.^1^^,^^2^ Common primary malignancies associated with uveal metastasis include carcinomas of the lung, breast, kidney, and gastrointestinal tract.^1^ Choroidal metastases typically manifest as amelanotic choroidal masses which can be differentiated from uveal melanoma based on high internal reflectivity on ultrasound and relatively rapid growth. These lesions are often multifocal and bilateral. Here, we describe a rare case of slowly progressive bilateral vision loss and retrobulbar pain from bilateral choroidal metastasis of a slow-growing neuroendocrine tumor of the lung. Delay in diagnosis of almost 2 years resulted in severe vision loss in both eyes. Ocular symptoms of eye pain and vision loss were the initial presentation of the malignancy; these symptoms improved after systemic therapy, and regression of the choroidal masses was seen.

## 2 Case presentation

A 64-year-old Hispanic male presented to the emergency room with gradually worsening blurry vision and pulsating retrobulbar pain in both eyes for 3–4 months. The patient reports being seen by an ophthalmologist in Mexico starting 1.5 years ago; he was informed he may have an eye tumor when last seen 5 months earlier. He had no other significant past medical or ocular history. On review of systems, he reported an episode of fever three months ago that resolved, chronic fatigue over the past three years, and chronic joint pain. He had no night sweats or weight loss. He is a non-smoker and has no cats or dogs at home but does work with cattle and horses. Lab work done in the emergency room was notable for a weakly positive QuantiFERON gold with normal chest x-ray. Serologic testing for Lyme disease, syphilis, ACE, ANA, rheumatoid factor, ESR, and HLA-B27 were all normal. Head CT revealed globe densities posteriorly in both eyes with no abnormality in the brain.

**Fig. 1:**
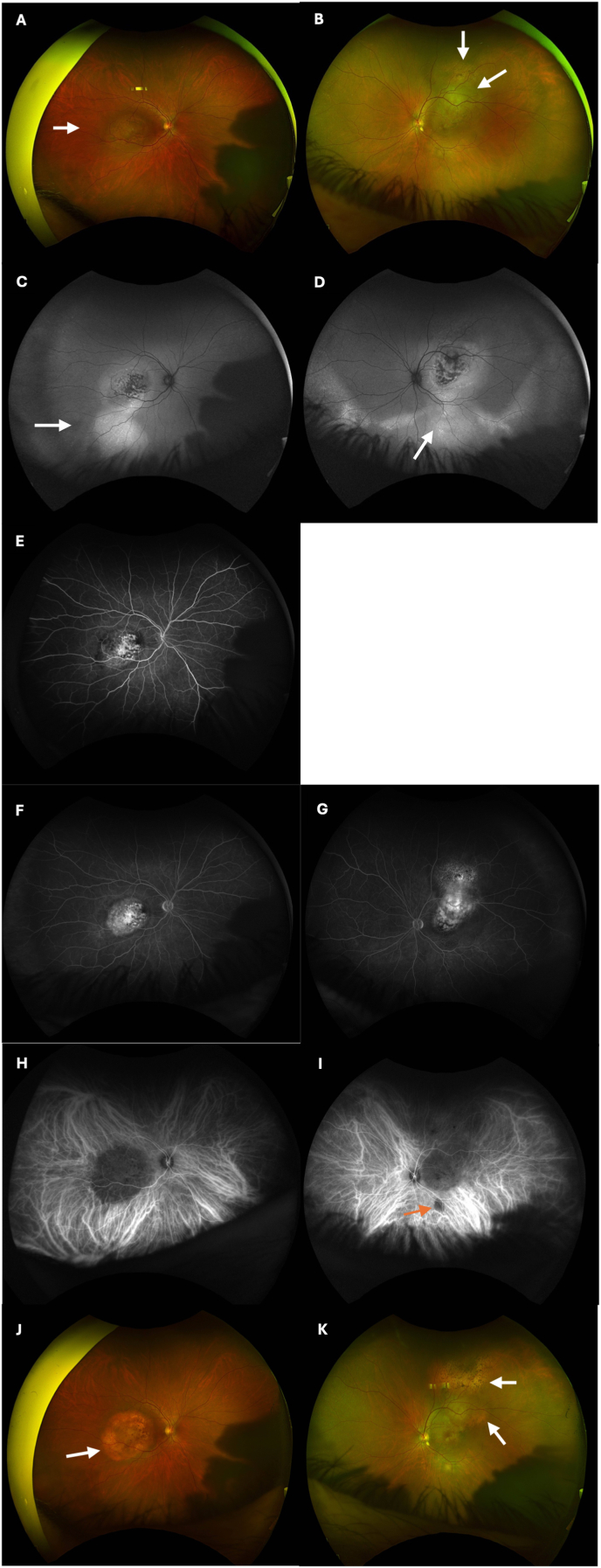
Ultra-widefield fundus image, fundus autofluorescence and angiograms of both eyes at initial presentation and after 3 months of systemic anti-cancer therapy. (A) Ultra-widefield fundus image of the right eye at presentation showing a large elevated amelanotic choroidal mass involving the entire macula with overlying pigmentary changes and subretinal fluid (arrow). (B) Ultra-widefield fundus image of the left eye at presentation showing a large elevated amelanotic choroidal mass involving the macula and extending superiorly beyond the supratemporal arcade. The mass appears multilobed (arrows). (C and D) Fundus autofluorescence images of the (C) right eye and (D) left eye at presentation showing hyper-autofluorescence of the macular tumor with mottled hypo-autofluorescence centrally in both eyes. Along the inferior edge of the mass, large areas of hyper-autofluorescence (arrows) are seen extending to the inferior periphery suggestive of guttering of subretinal fluid. (E) Early transit fluorescein angiogram at presentation of the right eye showing choroidal hypo-fluorescence in the macula in the region of the mass with mottled hyper-fluorescence within the mass. (F and G) Later transit fluorescein angiogram of the (F) right eye and (G) left eye showing hyper-fluorescence of the macular tumors in both eyes. (H and I) Indocyanine green angiogram obtained 3 weeks after presentation showing diffuse hypo-fluorescence of the macula tumor in the (H) right eye and diffuse hypo-fluorescence of the tumor in the (I) left eye which extends from the macula to superior mid-periphery. A separate small focus of hypo-fluorescence is observed inferior to the macula in the left eye (red arrow) which may represent a new site of tumor growth. (J and K) Ultra-widefield fundus images of the (J) right eye and (K) left eye 3 months after starting systemic therapy consisting of octreotide, Capecitabine and Temozolomide showing some atrophy of macular tumors in both eyes. (For interpretation of the references to color in this figure legend, the reader is referred to the Web version of this article.)

**Fig. 2:**
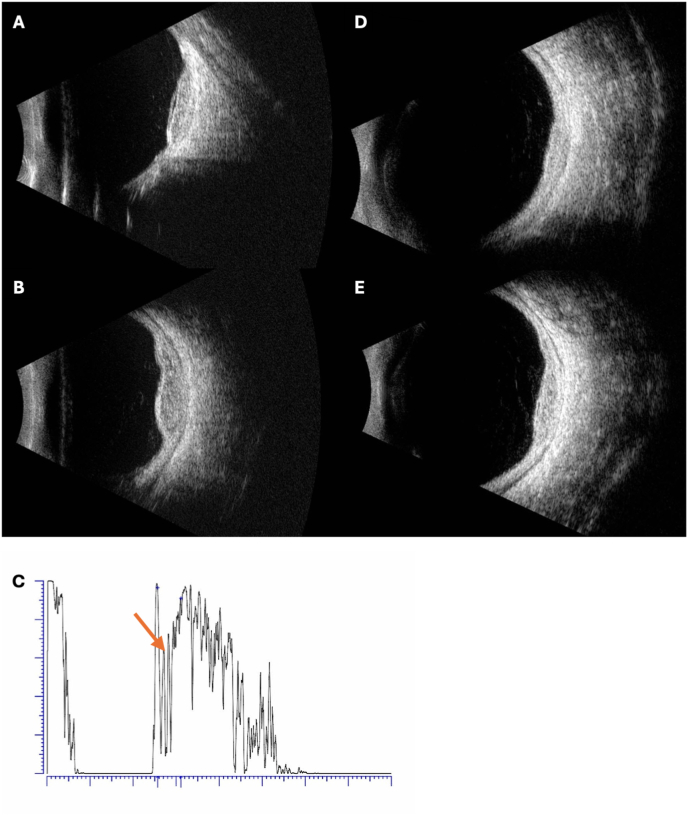
Ultrasound images of the choroidal tumor in both eyes at presentation and after 3 months of systemic therapy for metastatic neuro-endocrine tumor. (A) B-scan image of the right eye showing an elevated choroidal mass in the posterior pole at presentation. (B) B-scan image of the left eye showing an elevated, multilobed choroidal mass in the posterior pole at presentation. (C) A-scan image of the left eye at presentation showing low-medium internal reflectivity of the mass (orange arrow). (D and E) Follow-up B-scan ultrasound images of the (D) right eye and (E) left eye, demonstrating partial regression of the tumors 3 months after starting systemic therapy consisting of Octreotide, Capecitabine and Temozolomide. (For interpretation of the references to color in this figure legend, the reader is referred to the Web version of this article.)

**Fig. 3:**
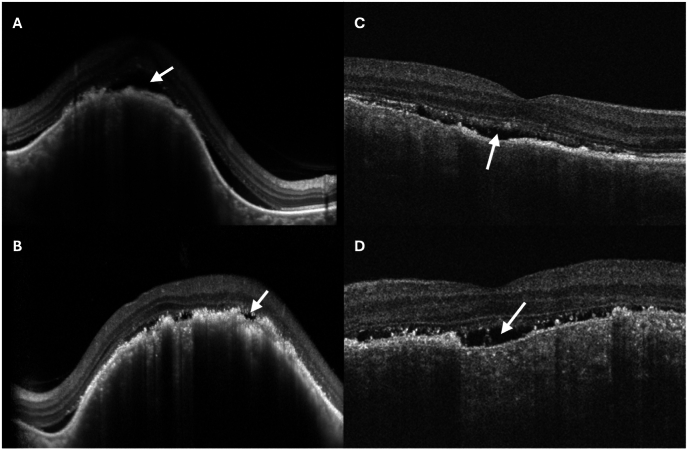
Macular optical coherence tomography (OCT) images of both eyes at presentation and at 3 months follow-up after systemic anti-cancer therapy. (A) The right macula at presentation showing focal elevation of the choroid with overlying subretinal fluid (arrow). (B) The left macula at presentation also showing focal choroidal elevation with overlying pockets of subretinal fluid (arrow). (C and D) Follow-up OCT of the macula of the right (C) eye and (D) left eye showing reduced elevation of the choroidal mass and decreased sub-retinal fluid (arrows) 3 months after systemic anti-cancer treatment.

On presentation to the ophthalmology clinic, visual acuity (VA) was 20/400 in both eyes, improving with pinhole to 20/200 in the right eye and no improvement in the left. Intraocular pressure was 10 mmHg bilaterally. Pupillary exam and extraocular movements were normal. Anterior segment examination was unremarkable except for mild cortical and nuclear sclerotic cataracts in both eyes. There were no cells in the anterior chamber or vitreous. Dilated fundus examination was notable for a large elevated amelanotic choroidal mass involving the macula in both eyes with overlying pigmentary changes and subretinal fluid (Fig. 1A and B). The lesion in the left eye had a multi-lobed appearance suggestive of a multifocal lesion that had coalesced. Fundus autofluorescence imaging showed hyper-autofluorescence of the lesions with central mottled hypo-autofluorescence in both eyes (Fig. 1C and D) with large areas of hyper-autofluorescence extending from the inferior tumor edge to the inferior periphery in both eyes, suggested of guttering of subretinal fluid. Ultrasound A and B-scan showed an elevated choroidal mass measuring 11.57mm × 10.20mm X 3.84mm thickness in the right eye and 14.70mm × 16.32mm X 3.84mm thickness in the left eye (Fig. 2A and B). The masses in both eyes had low-medium internal reflectivity (Fig. 2C) without evidence of scleral thickening or scleral invasion of the mass. No fluid was detected in the subtenon's space. Optical coherence tomography (OCT) confirmed focal elevation of the choroid with adjacent and overlying subretinal fluid in both eyes (Fig. 3A and B). Fluorescein angiography of the right eye showed choroidal hypo-fluorescence in the region of the mass with mottled hyper-fluorescence within the mass which increased in fluorescence in late transit suggestive of leakage and staining (Fig. 1E and F); the left eye showed similar findings in mid and late transit view (Fig. 1G). At this time, the differential diagnosis included metastasis given the bilateral presentation. Posterior scleritis was also considered given the patient's eye pain, but there was no fluid detected in subtenon's space or scleral thickening on ultrasound. A diagnosis also considered, but thought less likely, was choroidal granuloma given a weakly positive QuantiFERON gold. PET-CT scan was ordered to evaluate for malignancy, and while awaiting this, the patient was started on a trial of oral naproxen 500 mg twice daily for empiric treatment of posterior scleritis.

**Fig. 4:**
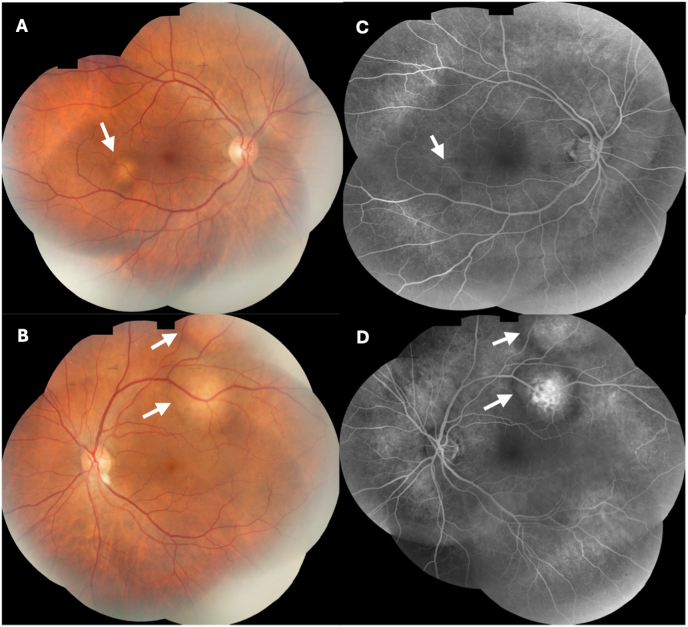
Fundus photograph and fluorescein angiogram of both eyes taken in Mexico 1.5 year prior to presentation. (A) Fundus photograph montage of the right eye. An arrow labels a small amelanotic lesion, less than a disc area in size, located infratemporal to the fovea. (B) Fundus photograph montage of the left eye with arrows showing two adjacent amelanotic choroidal lesions along the supratemporal arcade. (C) Fluorescein angiogram of the right eye shows mild hypo-fluorescence of the macula and infratemporal to the macula in the region of the choroidal lesion (arrow). (D) Fluorescein angiogram of the left eye shows hyper-fluorescence with rim of hypo-fluorescence of the two choroidal lesions (arrows) along the supratemporal arcade.

Three weeks later, the patient's vision was subjectively unchanged, but eye pain and irritation had worsened. The VA and fundus exam in the right eye remained stable, but VA in the left eye had decreased to counting fingers and the associated choroidal lesion was slightly larger. Given no improvement, naproxen was discontinued. During this visit, the patient brought fundus photograph and fluorescein angiography images of both eyes obtained in Mexico 1.5 years prior (Fig. 4A–D). There was marked growth of the choroidal lesions in both eyes over 1.5 years with corresponding progressive vision loss. Indocyanine green (ICG) angiography at this visit demonstrated hypo-fluorescence of the macular tumors and late diffuse leakage bilaterally (Fig. 1H and I); a second smaller area of hypo-fluorescence along the inferotemporal arcade of the left eye was noted which was suspicious for a new lesion (Fig. 1I).

A few days later, a PET-CT scan was completed; it demonstrated a hypermetabolic opacity in the left lower lobe of the lung concerning for an infectious or inflammatory etiology versus a low-grade malignancy. There were multiple bilateral pulmonary hilar lymph nodes with low-grade FDG activity. No overt abnormal FDG activity was seen within the head or neck. With this information, bilateral choroidal metastases were considered the likely diagnosis, and the patient was referred to medical oncology for further evaluation.

**Fig. 5:**
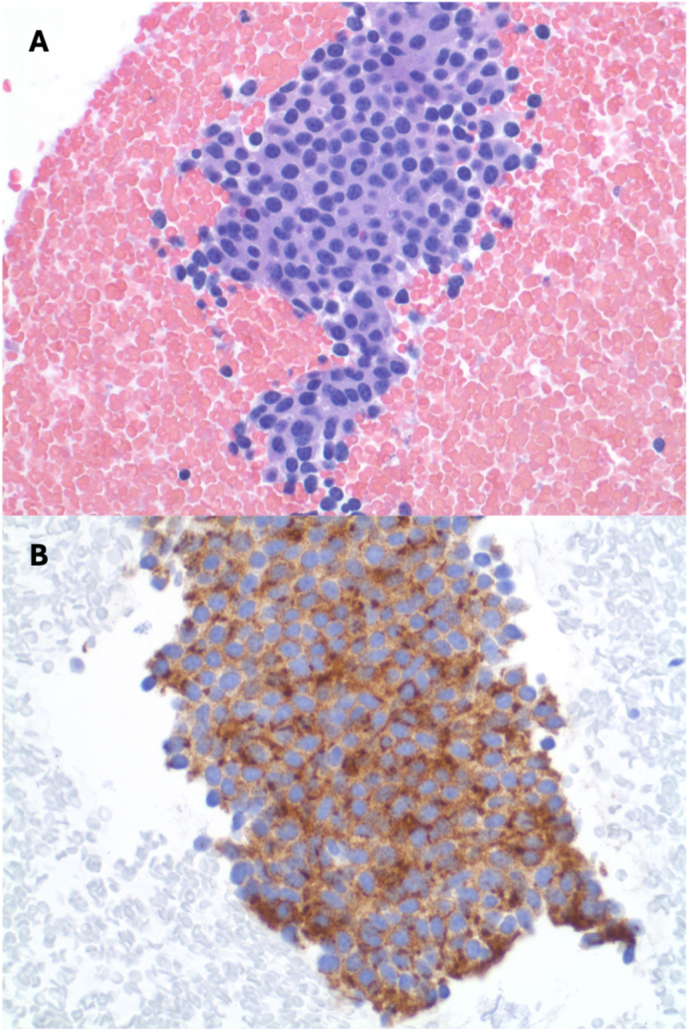
Histology of lung lesion biopsy. (A) H & E stain. (B) Chromogranin A stain.

Due to insurance issues, the patient was not seen by medical oncology or a pulmonary specialist until four months after his initial presentation to clinic. A bronchoscopy with bronchoalveolar lavage was negative for malignancy, but needle biopsies of the lesion in the left lower lobe of the lung and the pulmonary hilar lymph nodes showed chronic inflammation and probable slow growing neuroendocrine tumor that stained positive with Chromogranin A (Fig. 5). Medical oncology evaluated the patient and a diagnosis of neuroendocrine tumor of the left lower lobe lung with subcarinal/N2 disease was made (TX N2 M0/M1 grade 1 of likely pulmonary origin). Of note, the patient had no symptoms associated with carcinoid tumor of the lung, such as coughing, flushing, sudden warmth, or diarrhea. The patient was started on Octreotide injections and systemic chemotherapy with Capecitabine and Temozolomide. No systemic or local corticosteroid was used.

After three months of systemic therapy, the patient reported complete resolution of eye pain and improvement in vision in both eyes. Best corrected VA was 20/50 in the right and 20/200 in the left. Dilated fundus examination showed partial regression of the tumor in both eyes (Fig. 1J and K) with subretinal fluid decreased in both eyes on OCT (Fig. 3C and D). On ultrasound, the tumors in both eyes were smaller with the right measuring 9.94mm × 8.28mm x 2.36mm in thickness and the left measuring 15.01mm × 14.23mm X 3.10mm in thickness (Fig. 2D and E). Follow-up imaging showed reduction in size of the lung tumor. At the most recent follow-up visit after systemic therapy for six months, tumors in both eyes continued to decrease in size on ultrasonography; VA was 20/40 in the right and 20/100 in the left.

## 3 Discussion

In this case report, we describe a patient who presented with a four-month history of progressive bilateral vision loss and retrobulbar pain and was noted to have large bilateral choroidal masses involving the macula from metastasis of a slow growing neuroendocrine tumor of the lung. This patient had choroidal lesions that were observed for 1.5 years by different providers prior to presentation resulting in delay in diagnosis and treatment. After starting systemic cancer therapy, his vision loss improved and eye pain resolved, concurrent with decreases in tumor thickness and subretinal fluid in both eyes.

Choroidal metastases from neuroendocrine tumors are rare, likely reflecting the low prevalence of neuroendocrine tumors compared to other primary malignancies.^3^ Previous cases have been described in the literature.^4^, ^5^, ^6^ The largest case series of uveal metastasis from a neuroendocrine tumor was described by Harbour et al., where nine cases of carcinoid tumor with uveal metastasis were noted among a total of 410 consecutive cases of uveal metastasis (incidence 2.2%).^4^ While those cases share some characteristics to this patient, important differences exist. Similar to our case, five of the nine cases had no known primary malignancy at the time of eye diagnosis, the most common site of the primary malignancy was bronchus, and most patients did not have symptoms of carcinoid syndrome such as flushing, tachycardia, or diarrhea at the time of eye presentation.^4^ The choroid was the most common site of uveal metastasis, but other sites included the ciliary body and iris. All reported tumors were notably orange in color, which differs somewhat from that observed in our case. Choroidal metastases can vary in pigmentation from pale yellow to orange to dark-brown,^1^^,^^6^^,^^7^ and sometimes can be difficult to differentiate from choroidal melanoma. Interestingly, the choroidal masses in our patient had low-medium internal reflectivity on ultrasound, simulating a choroidal melanoma, albeit lacking the characteristic decrescendo. In contrast, previously reported cases of choroidal metastasis from a neuro-endocrine tumor had tumors with high to medium internal reflectivity on ultrasound A scan.^4^

Another notable feature of the current case is the significant retrobulbar eye pain in both eyes that developed with the relatively slow progressive choroidal tumor growth. The pathogenesis of posterior pole eye pain from choroidal masses in the absence of intraocular inflammation or anterior segment involvement is not well understood but has been hypothesized to originate from tumor necrosis, perineural invasion into the posterior ciliary nerves, or microscopic scleral involvement.^8^ These processes would lead to pain when any of the nerves originating from the trigeminal ganglion are involved, such as the long and short ciliary nerves. Ocular pain has been previously reported in cases of uveal melanoma and choroidal metastases from lung cancer, but there is only one published report of eye pain due to choroidal metastasis from a neuroendocrine tumor in one eye.^4^^,^^9^^,^^10^

Importantly, our patient had significant reduction in choroidal tumor size and improvement in vision and ocular pain with systemic treatment alone despite delays in diagnosis and treatment. Treatment of choroidal metastases from primary neuroendocrine tumors can involve systemic and targeted ocular therapy. In a large study of 520 eyes with uveal metastasis, local ocular treatment was delivered to 86% of patients.^1^ None of these patients were specifically noted to have neuroendocrine tumors. Previous reports of neuroendocrine tumors with ocular metastasis have required treatment of uveal metastasis with external beam radiotherapy, plaque radiotherapy, photocoagulation, photodynamic therapy, or local resection^4^^,^^11^^,^^12^ while the primary tumors were treated with systemic chemotherapy, surgical resection, wedge biopsy, or not treated in some cases.^4^ While local radiotherapy can be effective for local tumor control, it can result in vision loss from complications of radiation retinopathy or papillopathy for posterior pole tumors. Photodynamic therapy (PDT) has also demonstrated efficacy when choroidal metastases do not respond to systemic chemotherapy. One retrospective case series found tumor control in 78% of eyes treated with PDT with verteporfin.^11^ For our case where choroidal masses involved the macula in both eyes, systemic chemotherapy alone has been effective thus far in reducing the tumor size and reversing some of the associated vision loss. Either neoadjuvant therapy, which is given to shrink a tumor before the main treatment, or adjuvant therapy, which is delivered after the primary treatment if a tumor does not respond or regrows, can be considered if residual choroidal tumor remains or starts to regrow.

Another unique feature of our case is the long delay in diagnosis and treatment due to changes in providers and insurance barriers. These delays resulted in severe vision loss in both eyes as the tumors grew significantly over two years. This case exemplifies the importance of a thorough and expedited diagnostic workup and treatment. A more streamlined approach from the emergency room to ophthalmology to medical oncology would have minimized some of the delay. Despite these delays, our patient's vision improved gradually with systemic chemotherapy alone.

## 4 Conclusions

This case report describes a rare case of a patient presenting with a long history of progressive vision loss and retrobulbar pain in both eyes from metastasis of a slow growing, grade 1 neuroendocrine tumor of the lung. These eye tumors were observed by different providers for almost two years, resulting in delays in diagnosis, progressive growth of tumors, and severe vision loss in both eyes. The current systemic treatment, involving a combination of three agents, appears effective in treating these choroidal tumors as well as the primary lung tumor. Neuroendocrine tumors are relatively rare but can present initially as pigmented or amelanotic choroidal metastasis and vision loss, without concurrent hormone-associated symptoms that may be secondary to the primary tumor. The ophthalmologist can serve an important role in the management of such cases by beginning a systemic workup for malignancy and coordinating interdisciplinary care.

## CRediT authorship contribution statement

**Sharon H. Zhao:** Writing – original draft, Visualization, Investigation, Conceptualization. **Susanna S. Park:** Writing – review & editing, Visualization, Supervision, Resources, Project administration, Funding acquisition, Conceptualization.

## Funding source

This work was supported in part by the Barbara A. & Alan M. Roth, MD Endowed Chair of Discovery, Education & Patient Care in Visual Science (SSP) from the University of California Davis. The funding agency had no role in design or implementation of the study.

## Declaration of competing interest

The authors declare that they have no known competing financial interests or personal relationships that could have appeared to influence the work reported in this paper.
